# Dietary patterns and protein density associations with fat-free mass in a cross-sectional analysis of rural Iranian adults with overweight and obesity

**DOI:** 10.1038/s41598-026-49776-6

**Published:** 2026-04-24

**Authors:** Mohammad Ariya, Paul P. Fahey, Reza Homayounfar, Evan Atlantis

**Affiliations:** 1 School of Health Sciences, Western Sydney University, Campbelltown, NSW Australia; 2 Faculty of Nutrition and Food Technology, National Nutrition and Food Technology Research Institute, Shahid Beheshti University of Medical Sciences, Tehran, Iran; 3 Noncommunicable Diseases Research Center, Fasa University of Medical Sciences, Fasa, Iran

**Keywords:** Diseases, Endocrinology, Health care, Medical research, Risk factors

## Abstract

Fat-free mass (FFM), predominantly skeletal muscle, is associated with metabolic health, functional capacity, and longevity. This cross-sectional study investigated the association between dietary patterns, protein density, and Fat-Free Mass Index (FFMI) in 2,299 adults (799 men, 1,500 women) with overweight or obesity (body mass index ≥ 25 kg/m²) from the Fasa Adult Cohort Study in rural Iran. Dietary intake was assessed using a validated food frequency questionnaire, identifying two major patterns as Healthy and Unhealthy. Body composition was measured via bioelectrical impedance analysis. Multivariable linear regression assessed associations, while partitioning models adjusted for Fat Mass Index (FMI) to isolate relationships independent of adiposity. Initially, the Healthy pattern was positively associated with FFMI in men (β = 0.17, *P* = 0.003) but lost significance after adjusting for FMI (β = 0.09, *P* = 0.077). Conversely, the Unhealthy pattern showed a positive association with FFMI in men only after FMI adjustment (β = 0.10, *P* = 0.050). Protein Density was consistently associated with higher FFMI in both men (β = 0.01, *P* = 0.008) and women (β = 0.01, *P* = 0.047), independent of adiposity. Physical activity significantly mediated this relationship. These findings suggest that while broad dietary patterns may be confounded by body size, protein density is a robust, independent factor associated with muscle mass Interventions should prioritize protein density and physical activity to optimize body composition.

**Supplementary Information:**

The online version contains supplementary material available at 10.1038/s41598-026-49776-6.

## Introduction

The maintenance of Fat-Free Mass (FFM), comprised mainly of skeletal muscle, is fundamental to metabolic health, physical function, and overall longevity^1,2^. Currently, the global obesity epidemic, driven by an excess of Fat Mass (FM), has emerged as a paramount public health challenge^3^. The convergence of low FFM and high FM results in sarcopenic obesity, a phenotype that synergistically elevates the risk for cardiometabolic diseases, functional impairment, and all-cause mortality, often to a greater degree than either sarcopenia or obesity alone^4–6^. This condition is increasingly prevalent in Middle Eastern populations, including Iran, which are experiencing a rapid nutrition transition characterized by high rates of obesity and related non-communicable diseases^7,8^. However, standard metrics like Body Mass Index (BMI) are inadequate for distinguishing between FFM and FM^6,9^, creating a critical need for detailed body composition analysis to accurately characterize health risks in this specific population.

Among the modifiable lifestyle factors, dietary intake is a critical determinant of body composition^10,11^. Specifically, adequate dietary protein intake is widely established as a primary anabolic stimulus, providing the essential amino acids for muscle protein synthesis and the preservation of FFM, particularly in older adults or during weight loss^12,13^. Beyond single nutrients, a holistic dietary pattern approach is increasingly utilised to capture the complex interactions between foods and their cumulative effects on health^14^. This body of research consistently demonstrates that Prudent or Healthy dietary patterns, typically high in fruits, vegetables, whole grains, and lean protein sources, are associated with more favourable body composition, including higher muscle mass^15,16^. Conversely, Western or Unhealthy patterns, rich in refined grains, processed meats, and high-sugar foods, are linked to increased adiposity and a greater risk of sarcopenia^15–17^. Thus, both the anabolic potential of protein and the overall quality of the dietary pattern are essential components for maintaining FFM.

Furthermore, physical activity is recognized as a potent, independent stimulus for muscle protein accretion that acts synergistically with diet to modulate FFM, often by enhancing the anabolic response to nutritional stimuli^18^. Establishing the specific role of physical activity as a mediator for diet’s influence on FFM is important for people with overweight and obesity, as the synergistic impact of these behaviours on health outcomes likely exceeds their individual effects^19,20^.

Despite this established knowledge, significant gaps persist in the literature. For example, most studies exploring the relationship of protein intake or dietary patterns with body composition have been conducted in advanced economies, with research from the Middle East remaining limited^15,16,21^. Furthermore, existing research has predominantly focused on sarcopenia rather than the direct association of dietary patterns with FFM, and is often limited to older adults, single-gender cohorts, or specific dietary patterns, and rarely addressing individuals with overweight and obesity^15,16,22,23^. Perhaps most critically, a major methodological limitation obscures the true diet-FFM relationship. Given the established physiological coupling described by nutrient partitioning theories, where FM and FFM typically fluctuate in unison, it remains unclear whether observed associations are due to a direct effect of diet on FFM or are confounded by this strong correlation between FM and adiposity^24–26^. Addressing this confounding is particularly critical in populations with overweight and obesity. Because this demographic is the primary target for weight-loss interventions, the concurrent loss of FFM during caloric restriction is a major clinical concern that can exacerbate metabolic dysfunction and physical decline^27^. Therefore, clarifying the direct effect of diet on FFM (independent of FM) carries profound clinical relevance for designing interventions that successfully reduce adiposity while preserving vital muscle mass. Furthermore, although the interplay between diet and exercise is known^25,28^, the precise role of physical activity as a mediator in the specific relationship between different dietary patterns and FFM, especially after accounting for FM, is not well characterized.

In summary, there is a pressing need for detailed studies to clarify how diet and physical activity directly impact FFM, separate from FM, particularly in understudied populations. To address these gaps, this study pursued three main objectives. First, it explored the association between major dietary patterns, protein density, and FFM in a rural Iranian population with overweight and obesity. Second, to clarify the independent effect of diet, it assessed whether these associations remained significant after adjusting for FM. Finally, it explored the extent to which physical activity mediates the relationship between diet and FFM.

## Results

### Participant characteristics and selection bias

The initial dataset included 10,073 participants from the Fasa Adult Cohort Study (FACS). A total of 4,733 participants were excluded because they did not meet the BMI inclusion criterion (≥ 25 kg/m²) or were pregnant. After further excluding those with implausible energy intakes (greater than 4,700 or less than 800 kcal/day; *n* = 358), 4.982 eligible participants remained. Of this eligible cohort, 2,683 (53.6%) were missing FFM data due to a delay in device procurement. This left a final sample of 2,299 participants (799 men, 1500 women) with complete FFM measurements for analysis.

To assess for potential selection bias, baseline characteristics were compared between participants with FFM data (*n* = 2,299) and those without (*n* = 2,683), as detailed in Table 1. Participants included in the final analysis were, on average, slightly younger (47.4 vs. 49.2 years, *p* < 0.001), had more years of education (4.95 vs. 4.36 years, *p* < 0.001), and were more likely to be employed (44.2% vs. 38.7%, *p* < 0.001) than those excluded due to missing FFM data. The included group also reported slightly higher physical activity levels (40.0 vs. 39.3 MET-hours/week, *p* = 0.004). No significant differences were observed for gender, marital status, BMI, or waist circumference. The observed systematic difference in baseline variables, coupled with the fact that FFM data were systematically absent for the first half of enrolled participants due to the device procurement delay, suggests results are only generalisable to this slightly younger, slightly more educated population in FACS.

**Table 1:** Characteristics of Participants with and Without Fat-Free Mass (FFM) Measurements.

		FFM Measurements				*P*-value
		Without FFM Data (*n* = 2,683)		With FFM Data (*n* = 2,299)		
		Number	Percent	Number	Percent	
Gender	Male	939	35.0%	799	34.8%	0.857
	Female	1744	65.0%	1500	65.2%	
Marital Status	Single/Widow/Divorced	313	11.7%	236	10.3%	0.115
	Married	2370	88.3%	2063	89.7%	
Occupational Status	Without Job	1642	61.3%	1280	55.8%	< 0.001
	Having a Stable Job	1038	38.7%	1015	44.2%	

	Mean	SD	Mean	SD	*P*-value
Age (years)	49.18	9.40	47.44	9.15	< 0.001
Height (Cm)	160.50	8.68	160.22	8.84	0.267
Weight (Kg)	75.33	11.48	75	11.06	0.298
WC (cm)	101	8.98	100.83	8.77	0.483
BMI (kg/m^2^)	29.19	3.47	29.18	3.44	0.944
Education (Years)	4.36	3.72	4.95	3.84	< 0.001
Physical Activity (MET-hours/week)	39.25	10.01	40.04	9.40	0.004
Socio-economic Status	0.14	2.13	0.23	2.17	0.138

BMI, body mass index; FFM, fat-free mass; MET, metabolic equivalent of task; WC, waist circumference.

### Characteristics by fat-free mass index (FFMI) tertile

Baseline characteristics of the analytical sample, stratified by gender and Fat-Free Mass Index (FFMI) tertiles, are presented in Table 2. In both men and women, participants in the highest FFMI tertile (T3) were significantly younger (*p* < 0.001) and had a higher weight, waist circumference, and BMI (all *p* < 0.001) compared to those in the lowest tertile (T1). Among men, higher FFMI tertile was associated with more years of education (*p* = 0.010) and a greater likelihood of being employed (*p* = 0.001). After adjusting for age, the associations with education, physical activity, and socioeconomic status became significant in both genders. Furthermore, while total energy intake did not differ significantly across FFMI tertiles for either men or women, notable dietary differences emerged. Among men, a higher FFMI tertile was significantly associated with greater absolute protein intake (*p* < 0.001) and higher protein density (*p* = 0.016). After adjusting for age, the association for absolute protein intake remained significant (*p* = 0.002), whereas the association for protein density was attenuated (*p* = 0.064). Conversely, among women, there were no statistically significant differences in absolute protein intake, protein density, or total energy intake across the FFMI tertiles.

**Table 2:** Comparison of Characteristics According to Fat-Free Mass Index (FFMI) Tertiles in Men and Women.

		Tertile of FFM Measurements						*P*-value	Age adjusted *P*-value^*^
		T1		T2		T3			
		Number	Percent	Number	Percent	Number	Percent		
**Male**		*N* = 266		*N* = 267		*N* = 266			
Marital Status	Single/Widow/Divorced	7	2.6%	7	2.6%	5	1.9%	0.808	0.371
	Married	259	97.4%	260	97.4%	261	98.1%		
Occupational Status	Without Jobs	45	17.0%	23	8.6%	21	7.9%	0.001	0.541
	Having a Stable Job	220	83.0%	243	91.4%	245	92.1%		
**Female**		*N* = 500		*N* = 500		*N* = 500			
Marital Status	Single/Widow/Divorced	85	17.0%	70	14.0%	62	12.4%	0.110	0.691
	Married	415	83.0%	430	86.0%	438	87.6%		
Occupational Status	Without Jobs	394	78.8%	399	79.8%	398	79.9%	0.891	0.733
	Having a Stable Job	106	21.2%	101	20.2%	100	20.1%		

		Mean	SD	Mean	SD	Mean	SD	*P*-value	Age adjusted *P*-value^*^
**Male**		*N* = 266		*N* = 267		*N* = 266			
Age (years)		51.15	9.94	46.42	8.75	45.28	8.17	< 0.001	-
Weight (Kg)		75.54	6.79	79	7.44	89.06	11.78	< 0.001	0.273
WC (cm)		96.29	5.94	97.05	6.40	103.4	9	< 0.001	0.207
BMI (kg/m^2^)		26.36	1.11	27.63	1.53	30.81	3.49	< 0.001	0.409
Education (Years)		5.76	4.57	6.40	4.16	6.87	3.90	0.010	0.002
Physical Activity (MET-hours/week)		42.25	13.12	43.18	12.99	44.07	13.58	0.282	0.003
Socio-economic Status		0.77	2.53	0. 98	2.61	1.31	2.75	0.055	0.003
Total Energy Intake (kcal/day)		2797.97	859.54	2726.90	801.88	2860.19	880.41	0.193	0.200
Absolute Protein Intake (g/day)		111.29	36.26	119.15	48.91	128.43	48.24	< 0.001	0.002
Protein Density (% of energy from protein)		17.55	8.62	18.67	9.19	19.97	11.04	0.016	0.064
**Female**		*N* = 500		*N* = 500		*N* = 500			
Age (years)		49.03	9.63	47.24	8.85	45.77	8.37	< 0.001	-
Weight (Kg)		67	6.96	69.54	7.59	78.55	10.43	< 0.001	0.249
WC (cm)		98.33	6.72	100.12	7.89	107.09	9.75	< 0.001	0.208
BMI (kg/m^2^)		27.15	1.83	29.04	2.29	32.82	3.61	< 0.001	0.446
Education (Years)		3.98	3.64	4.17	3.11	4.49	3.40	0.053	0.001
Physical Activity (MET-hours/week)		38.49	5.73	38.51	5.71	38.14	6.11	0.530	0.001
Socio-economic Status		−0.27	1.83	−0.17	1.70	−0.12	1.68	0.386	< 0.001
Total Energy Intake (kcal/day)		2639.74	773.30	2648.79	824.60	2690.85	862.16	0.576	0.786
Absolute Protein Intake (g/day)		83.04	28.27	83.34	28.91	86.46	31.42	0.129	0.397
Protein Density (% of energy from protein)		13.44	5.62	13.69	6.01	14.22	7.16	0.141	0.308

FFMI, fat-free mass index; BMI, body mass index; WC, waist circumference; MET, metabolic equivalent of task.

### Dietary pattern identification

Principal Component Analysis (PCA) revealed two major dietary patterns, whose defining factor loadings are restricted to two patterns (Table 3). Visual inspection suggested the first pattern appeared to be healthier and the second pattern less healthy. The diet designated as Healthy dietary pattern was characterised by high factor loadings for fruits (0.64), fish (0.61), nuts (0.59), dairy products (0.50), and vegetables (0.49), with additional positive loadings for olive oil, whole grains, and red meat. The pattern designated as Unhealthy dietary pattern showed high loadings for sweets and desserts (0.64), saturated fats (0.62), sweet drinks (0.59), refined grains (0.51), and processed meats (0.50). Notably, saturated fats demonstrated a negative loading on the Healthy pattern and a positive loading on the Unhealthy pattern. Food groups that did not load significantly onto either pattern or exhibited substantial cross-loading (difference < 0.15), and were therefore iteratively excluded from the final models, were legumes and soy, chicken, egg, and pickles.

**Table 3:** Factor Loading and Scattering Matrices Expressed for Dietary Patterns Determined by The Factor Loading Method.

Food Groups	Healthy Dietary Pattern	Unhealthy Dietary Pattern
Fruits	0.64	-
Fish	0.61	-
Nuts	0.59	-
Dairy Products	0.50	-
Vegetables	0.49	0.20
Visceral Meat (miscellaneous animal-based foods)	0.49	0.32
Olive or Olive Oil	0.48	−0.22
Dried fruits	0.46	-
Whole Grains	0.46	-
Natural Fruit Juice	0.45	-
Red Meat	0.43	0.24
Liquid Oil	0.42	−0.36
Pizza	0.40	-
Cheese	0.34	-
Sweets and Desserts	-	0.64
Saturated Fats	−0.25	0.62
Sweet or Soft Drinks	0.29	0.59
Refined Grains	-	0.51
Processed Meats	-	0.50
Tea and coffee	-	0.43
Snacks (Puffs, Chips, and so on)	-	0.35
Salt	-	0.34
Sauces and Condiments		0.29

### Association of dietary variables with FFMI

The factor scores for both the Healthy and Unhealthy dietary patterns were confirmed to approximate a standardized normal distribution, demonstrating their suitability for use as continuous variables in the multivariable linear regression models (Supplementary Fig. 1). Multivariable Associations between dietary patterns and FFMI are summarized in Table 4. Among men, both the Healthy dietary pattern (Model A3: β = 0.17, 95% CI [0.06, 0.29], *p* = 0.003) and Protein Density (Model A3: β = 0.01, 95% CI [< 0.01, 0.03], *p* = 0.009) were positively associated with FFMI in fully adjusted models. The Unhealthy dietary pattern was initially associated with higher FFMI (Model A1: β = 0.16, 95% CI [0.04, 0.28], *p* = 0.006), but this association was no longer statistically significant after full adjustment (Model A3: β = 0.07, 95% CI [−0.05, 0.19], *p* = 0.250).

**Table 4:** Multivariable Linear Regression Analysis of the Association between Dietary Patterns, Protein Density, and Fat-Free Mass Index (FFMI).

	Fat-Free Mass Index (FFMI)								
	Model A1 (Crude)		Model A2 (Basic Adjusted)		Model A3 (Fully Adjusted)				
	β (95% CI)	*P*-value	β (95% CI)	*P*-value	β (95% CI)		*P*-value		
**Male**									
Healthy Dietary Pattern	0.23 (0.12, 0.34)	< 0.001	0.17 (0.06, 0.27)	0.001	0.17 (0.06, 0.29)		0.003		
Unhealthy Dietary Pattern	0.16 (0.04, 0.28)	0.006	0.07 (−0.04, 0.18)	0.232	0.07 (−0.05, 0.19)		0.250		
Protein Density	0.01 (< 0.01, 0.2)	0.010	0.01 (< 0.01, 0.03)	0.006	0.01 (< 0.01, 0.03)		0.009		
**Female**									
Healthy Dietary Pattern	0.02 (−0.05, 0.10)	0.575	−0.003 (−0.08, 0.07)	0.947	0.01 (−0.07, 0.10)		0.759		
Unhealthy Dietary Pattern	0.12 (0.05, 0.20)	< 0.001	0.07 (−0.001, 0.14)	0.053	0.06 (−0.01, 0.13)		0.127		
Protein Density	0.01 (0.00, 0.02)	0.043	0.01 (< 0.01, 0.03)	0.021	0.01 (< 0.01, 0.02)		0.038		

Model A2: Adjusted for age, total energy intake (kcal/day) and physical activity (MET-hours/week); Model A3: Additionally adjusted for education (years), marital status, occupational status, and socio-economic status.

Among women, Protein Density showed a statistically significant positive association with FFMI in the fully adjusted model (Model A3: β = 0.01, 95% CI [< 0.01, 0.02], *p* = 0.038). The Unhealthy dietary pattern showed a statistically significant association in the crude model (Model A1: β = 0.12, 95% CI [0.05, 0.20], *p* < 0.001), but was attenuated after full adjustment (Model A3: β = 0.06, 95% CI [−0.01, 0.13], *p* = 0.127). The Healthy dietary pattern was not statistically significantly associated with FFMI in any primary model.

In the secondary partitioning analyses (Supplementary Table 1), which adjusted for Fat Mass Index (FMI) to isolate associations independent of adiposity, these relationships were altered. Among men, the association with the Healthy pattern was attenuated to non-significance (Model B3: β = 0.09, *p* = 0.077), while positive associations with the Unhealthy pattern (Model B3: β = 0.01, *p* = 0.050) and Protein Density (Model B3: β = 0.01, *p* = 0.008) persisted. Among women, the positive association with Protein Density also remained after FMI adjustment (Model B3: β = 0.01, *p* = 0.047).

### Mediation analyses by physical activity

Mediation analysis examining the role of physical activity in the association between dietary variables and FFMI are presented in Table 5. In the primary models (Table 5), physical activity did not significantly mediate the association between any of the dietary variables (Healthy pattern, Unhealthy pattern, Protein Density and FFMI in the fully adjusted models (Model C3) for either men or women. All 95% bootstrap confidence intervals for the indirect effects included zero.

**Table 5:** Mediation Analysis of Physical Activity (MET-hours/week) on the Association Between Dietary Patterns and Fat-Free Mass Index (FFMI).

	Fat-Free Mass Index (FFMI)						
	Direct Effect				Indirect Effect(s)		
	β	SE	95% CI	*P*-value	β	Boot SE	95% Boot CI
**Male/Healthy Dietary Pattern**							
Model C1 (Crude)	0.24	0.05	0.13, 0.34	0.000	−0.007	0.005	−0.01-0.0006
Model C2 (Basic Adjusted)	0.17	0.05	0.06, 0.27	0.001	−0.007	0.005	−0.01-0.0006
Model C3 (Fully Adjusted)	0.17	0.05	0.06, 0.29	0.002	−0.004	0.004	−0.01-0.002
**Female/Healthy Dietary Pattern**							
Model C1 (Crude)	0.02	0.04	−0.05, 0.10	0.558	−0.001	0.003	−0.008-0.005
Model C2 (Basic Adjusted)	−0.00	0.04	−0.08, 0.07	0.946	−0.0008	0.003	−0.008-0.005
Model C3 (Fully Adjusted)	0.01	0.04	−0.07, 0.10	0.751	−0.0003	0.001	−0.003-0.002
**Male/Unhealthy Dietary Pattern**							
Model C1 (Crude)	0.16	0.06	0.04, 0.28	0.006	0.002	0.003	−0.003-0.01
Model C2 (Basic Adjusted)	0.07	0.05	−0.04, 0.18	0.231	0.002	0.003	−0.003-0.01
Model C3 (Fully Adjusted)	0.07	0.06	−0.04, 0.19	0.239	−0.0009	0.003	−0.008-0.004
**Female/Unhealthy Dietary Pattern**							
Model C1 (Crude)	0.12	0.03	0.05, 0.20	0.000	−0.0002	0.004	−0.008-0.008
Model C2 (Basic Adjusted)	0.07	0.03	−0.00, 0.14	0.052	0.0003	0.004	−0.007-0.009
Model C3 (Fully Adjusted)	0.06	0.03	−0.01, 0.13	0.117	0.0003	0.001	−0.002-0.003
**Male/Protein Density**							
Model C1 (Crude)	0.01	0.00	0.00, 0.02	0.012	0.0005	0.0005	−0.0003-0.001
Model C2 (Basic Adjusted)	0.01	0.00	0.00, 0.03	0.006	0.0008	0.0008	−0.0005-0.002
Model C3 (Fully Adjusted)	0.01	0.00	0.00, 0.03	0.009	0.0004	0.0006	−0.0005-0.001
**Female/Protein Density**							
Model C1 (Crude)	0.01	0.00	0.00, 0.02	0.043	0.00	0.0002	−0.0004-0.0006
Model C2 (Basic Adjusted)	0.01	0.00	< 0.01, 0.03	0.020	0.00	0.0007	−0.001-0.001
Model C3 (Fully Adjusted)	0.01	0.00	< 0.01, 0.02	0.032	0.0001	0.0004	−0.0006-0.001

FFMI, fat-free mass index; MET, metabolic equivalent of task; SE, standard error; Boot CI, bootstrap confidence interval. Model C2: Adjusted for age and total energy intake (kcal/day); Model C3: Additionally adjusted for education (years), marital status, occupational status, and socio-economic status.

In contrast, in the secondary partitioning mediation models (Supplementary Table 2), which adjusted for FMI, several indirect effects became significant. Physical activity was found to significantly mediate the association between Protein Density and FFMI in adjusted models for both men (Model D3: Indirect Effect β = 0.001, 95% Boot CI [0.00, 0.003]) and women (Model D2: Indirect Effect β = 0.001, 95% Boot CI [0.0003, 0.003]). A significant negative mediation was observed for the Healthy pattern in both men (Model D2: Indirect Effect β = −0.01, 95% Boot CI [−0.02, −0.001]) and women (Model D2: Indirect Effect β = −0.007, 95% Boot CI [−0.01, −0.001]). For the Unhealthy pattern, physical activity significantly mediated the association with FFMI in women (Model D2: Indirect Effect β = 0.009, 95% Boot CI [0.001, 0.02]) but not for men.

## Discussion

This study provides novel insights into the relationships between diet, adiposity, and FFM in a rural Iranian population with high rates of overweight and obesity. Our findings, which directly address our three research aims, reveal a complex picture dominated by two key themes: the robust, independent role of protein density and the critical importance of accounting for FM to avoid confounding. Specifically, we found that: (1) a Healthy dietary pattern was associated with higher FFMI in men only before adjusting for FMI; (2) an Unhealthy pattern was paradoxically associated with higher FFMI in men after this adjustment; and (3) higher protein density was consistently associated with higher FFMI in both men and women, independent of adiposity, with physical activity significantly mediating this association.

A central methodological finding is that failing to adjust for FMI can profoundly confound the diet-FFMI relationship. The positive association between the Healthy dietary pattern and FFMI in men was completely attenuated to non-significance in our partitioning models. This suggests that the initial association was a “loading effect” of overall energy intake and body size, rather than a specific anabolic association with the diet itself, and once the shared variance with FMI was accounted for, no specific benefit for FFM remained. This interpretation is strongly supported by previous findings in the FACS cohort, where higher adherence to a Healthy dietary pattern was linked to greater energy intake and, paradoxically, higher body weight and FM^29^. Similarly, another study identified that individuals with “high intake” profiles (consuming more of both healthy and unhealthy foods) had significantly higher energy intake compared to “low intake” groups^30^. Once we adjusted for FMI in the partitioning models, the association between the Healthy pattern and FFMI disappeared, suggesting that in this specific rural context, the Healthy pattern is not independently associated with an anabolic advantage for muscle mass independent of body size. This aligns with prior work in the same cohort, which found that associations between the Mediterranean diet and body composition markers often lost significance after adjusting for energy intake, highlighting the confounding role of total calories^31^.

Unexpectedly, the Unhealthy dietary pattern (rich in sweets, saturated fats, and refined grains) showed a positive association with FFMI in men only after adjusting for FMI in the partitioning models. While typically linked to sarcopenia^32^, this finding suggests that in the context of this rural population, high intake of energy-dense foods is associated with the maintenance of muscle mass. This association may be partly explained by insulin-mediated anabolism. In other words, high intake of refined carbohydrates and fats is known to stimulate insulin secretion, a potent anabolic hormone that inhibits muscle protein breakdown and promotes nutrient storage^33^. Crucially, however, this association is highly susceptible to reverse causality. Individuals with a higher pre-existing fat-free mass possess greater resting metabolic rates, which inherently drives higher total energy demands^34^. In a rural setting undergoing rapid nutritional transition, this increased energy requirement is frequently met through the consumption of highly accessible, energy-dense foods that characterize the Unhealthy pattern^35^. Therefore, a larger muscle mass likely drives the consumption of the Unhealthy pattern, rather than the diet itself promoting muscle accretion. In the absence of FMI adjustment, this relationship was masked, but partitioning out the FMI revealed the underlying association with high energy intake.

However, this apparent preservation comes at the cost of increased metabolic risk. Crucially, while bioelectrical impedance analysis captures total FFM, it cannot differentiate muscle quantity from muscle quality^36^. The observed higher FFMI may be confounded by myosteatosis (intramuscular adipose tissue infiltration), a condition frequently exacerbated by unhealthy dietary patterns and linked to functional decline despite preserved mass^37^. Moreover, as noted in previous FACS analyses, animal protein (often a component of mixed or unhealthy patterns in this region) was associated with lower abdominal obesity but mixed metabolic outcomes^38^. This mirrors the “obesity paradox” observed in other populations, where higher BMI and energy intake are associated with protection against muscle wasting in older adults but exacerbate cardiometabolic risk^21,39^. Similarly, research in other Asian populations found that “grain-meat” patterns were associated with preserved muscle but higher rates of metabolically healthy obesity, a phenotype that often transitions to metabolic disease over time^40,41^.

A key finding of this study is the robust, positive association between Protein Density and FFMI in both men and women, which persisted even after adjusting for FMI. Our methodological selection of protein density was crucial here. In populations with elevated adiposity, adjusting protein intake for total body weight frequently underestimates true nutritional adequacy because the metric’s denominator is artificially inflated by excessive, metabolically less-active FM^42^. By utilising Protein Density, we successfully isolated the anabolic quality of the diet relative to total energy demands, avoiding the confounding influence of body size. This also supports the “protein leverage” hypothesis and the established role of amino acids in stimulating the Mammalian Target of Rapamycin (mTOR) pathway associated with muscle protein synthesis^13^.

Our results are consistent with large-scale data from the UK Biobank, which demonstrated that higher protein intake was linearly associated with greater FFM and grip strength across a wide age range^43^. Furthermore, expert groups have argued that adults may require higher protein intake than the current Recommended Dietary Allowance (RDA) to maintain muscle mass, a view supported by findings that higher protein intake prevents functional decline^10,12,44^. By demonstrating this link independent of FMI, our study suggests that Protein Density is a more critical correlate of muscularity than the overall dietary pattern in this rural population. Although the unstandardized β-coefficient of 0.01 appears numerically small, it reflects the change in FFMI per single percentage point increase in protein energy. Consequently, a clinically realistic 10% increase in dietary protein density (e.g., from 10% to 20% of total daily energy) corresponds to a 0.10 kg/m² higher FFMI, a shift that is biologically meaningful for mitigating age-related muscle loss at the population level. This is particularly relevant for women in our study, where Protein Density was the sole dietary factor associated with protection against low muscle mass, consistent with findings that women may have different protein utilization efficiencies compared to men^45^.

Our mediation analysis offers novel insights into the behavioural pathways associated with diet and body composition. In the primary models, the substantial variance associated with overall adiposity and body size likely masked specific behavioural pathways. However, in the partitioning models (adjusted for FMI), statistically isolating the lean tissue compartment revealed that physical activity emerged as a significant mediator for Protein Density. Crucially, because mediation analysis applied to cross-sectional data cannot establish temporal precedence, this result does not imply a directional causal pathway. Rather, it suggests that the statistical association between Protein Density and FFMI is partly explained by shared variance with physical activity. This finding likely reflects a clustering of health-promoting behaviours. This synergistic relationship aligns with recent reviews indicating that nutritional stimuli are most effective when sensitized by mechanical loading from exercise^28^.

Furthermore, we observed a significant negative mediation (in the partitioning model) for the Healthy pattern in both genders. In rural Iran, this may reflect a socio-occupational shift: individuals with higher socioeconomic status and greater access to diverse healthy foods may simultaneously transition away from physically demanding agricultural labour toward more sedentary occupations^46^. Conversely, in the partitioning model, physical activity positively mediated the relationship between the Unhealthy pattern and FFMI in women, but not in men. This sex-specific finding may highlight the energy demands of agricultural or domestic labour roles predominantly performed by rural women in this region^47^. Women in these roles may rely on energy-dense foods (Unhealthy pattern) to meet caloric needs, with high physical activity preserving muscle mass. These interpretations remain speculative; physical activity in rural settings is often occupation-driven, and differential measurement of activity types by gender may influence these pathways^48,49^. Ultimately, the relationship between diet quality and physical activity is complex; recent interventions have shown that while dietary changes can induce weight loss, the addition of physical activity is crucial for optimizing cardiometabolic health^50^.

The attenuation of dietary associations after adjusting for FMI highlights the methodological necessity of partitioning models in obesity research. Indeed, in states of obesity, the hydration of FFM can be altered by extracellular water expansion in adipose tissue, potentially inflating FFM estimates derived from bioelectrical impedance^51^. By adjusting for FMI, we aimed to control for this “bystander” effect. The persistence of the Protein Density-FFMI association in these adjusted models suggests a robust biological association, whereas the attenuation of the Healthy pattern association suggests those previous findings may have been confounded by the generally larger body size of individuals consuming those diets.

The results of this study should be interpreted in the context of several limitations. First, the cross-sectional design precludes causal inference; we cannot determine whether dietary patterns and higher protein density correlate with maintained FFM or if individuals with higher muscle mass consume more food due to higher metabolic demands^52^. Second, dietary intake was assessed using an Food Frequency Questionnaire (FFQ), which is subject to recall bias, although this method has been validated for the FACS population^53^. The associations identified between dietary patterns and FFMI in this study are based on a specific subset of the rural Iranian population. Due to limited geographic and demographic scope, these findings may not be representative of the broader rural population across Iran.

Furthermore, the factor analysis revealed unexpected loadings that complicate interpretation. The Healthy pattern includes a positive loading for Pizza (0.40), while the Unhealthy pattern shows positive loadings for Tea and Coffee (0.43) and Vegetables (0.20). Rather than methodological errors, these anomalies likely reflect specific cultural dietary behaviours in rural Fasa; for instance, Pizza in this region is predominantly a homemade dish consumed by higher socioeconomic groups who also consume fruits and nuts. Consequently, pizza clusters statistically with these healthy indicators. Conversely, while tea consumption is ubiquitous, it is traditionally consumed with high amounts of sugar (sugar cubes) in the Iranian population, which strongly drives its clustering alongside sweets, saturated fats, and refined grains in the Unhealthy pattern^29,38^.. Similarly, while vegetables strongly characterize the Healthy pattern (loading = 0.49), their weak positive loading on the Unhealthy pattern (0.20) likely reflects their ubiquitous consumption as side dishes or in traditional stews cooked with high amounts of fat. To ensure these anomalies did not skew our results, we conducted a sensitivity analysis by removing pizza and these weak cross-loading items from the PCA; the core factor structure and their subsequent associations with FFMI remained robust, leading us to retain the original variables to accurately reflect the population’s true dietary habits.

In conclusion, this study successfully achieved its aims, demonstrating that the apparent positive associations of a Healthy dietary pattern on FFM were largely confounded by adiposity, while protein density was a consistent, independent predictor. The paradoxical association of an Unhealthy pattern with FFMI highlights the complex interplay of energy intake and body composition. For public health and clinical practice in this region, these findings suggest a prioritized focus on ensuring adequate protein intake and promoting physical activity may be more effective for preventing sarcopenic obesity than recommending broad dietary patterns alone.

## Methods

### Study design and participants

This cross-sectional analysis utilised data from the FACS, a component of the Prospective Epidemiological Research Studies in Iran (PERSIAN) cohort^7^. The FACS enrolled 10,073 adults aged 35–70 residing in the rural Sheshdeh region of Fasa, Southern Iran, starting from 2014. The study protocol was approved by the Institutional Review Board of Fasa University of Medical Sciences (IR.FUMS.REC.1400.139) and conformed to the Declaration of Helsinki. All participants provided written informed consent^7^.

### Dietary assessment

Dietary intake was assessed at baseline using a validated 125-item semi-quantitative FFQ administered by trained interviewers^7,53^. Participants reported the frequency (daily, weekly, monthly, or yearly) and portion size of each food item consumed during the previous year. Total daily energy (kcal/day) and nutrient intakes were derived from the FFQ using an Iranian-adapted version of Nutritionist IV software^7^, with participants reporting implausible energy intakes (< 800 or > 4,700 kcal/day) then being identified and excluded. Protein Density was calculated as the percentage of total energy intake derived from protein, using the formula: (Protein intake [g/day] × 4 kcal/g)/Total energy intake [kcal/day] × 100. This specific metric was selected over absolute intake (g/day) or weight-adjusted intake (g/kg/day) due to the distinct anthropometric profile of our cohort. Absolute intake is heavily confounded by total energy consumption^42,54^, while weight-adjusted metrics are systematically skewed in populations with overweight and obesity because the high proportion of metabolically less active fat mass artificially inflates the denominator^55^. Protein density effectively isolates the macronutrient quality of the diet independent of total caloric intake and body size^42^.

Moreover, dietary patterns were derived via PCA with Varimax orthogonal rotation, following aggregation of food items from the FFQ into 27 nutritionally similar food groups. Daily intakes were transformed using the natural logarithm of (g/day + 0.1) to address skewness and zero intakes. The number of factors retained was determined by interpretability and scree plot inspection. Food groups with absolute factor loadings above 0.2 or below − 0.2 were considered significant contributors. Cross-loading food groups (difference < 0.15 between main and secondary pattern) were removed iteratively to optimize factor stability and interpretability. This iterative exclusion process ensures that the final retained factors represent clearly delineated dietary behaviours, allowing for reproducible identification of distinct patterns. Additionally, a sensitivity analysis was performed by iteratively excluding counterintuitive variables to ensure that these cultural anomalies did not artificially drive the factor structure or alter the final regression outcomes. Final factor scores for both Healthy and Unhealthy dietary patterns were computed for each individual by summing the daily intake (g) of the food groups identifying each pattern.

### Body composition and anthropometry

Anthropometric measurements were performed by trained personnel according to the FACS protocol^7^. Height was measured to the nearest 0.1 cm using a stadiometer while participants were standing without shoes. Waist Circumference (WC) was measured at the narrowest point of the waist, to the nearest 0.1 cm^7,29^.

Body composition was assessed in a fasted state using a multi-frequency Bioelectrical Impedance Analysis (BIA) device (Tanita BC-418, Tanita, Tokyo, Japan)^29^. This device provided estimates of total body FM and FFM in kilograms.

BMI was calculated as weight (kg) divided by height in meters squared (m²). FMI and FFMI were subsequently calculated by dividing the respective mass component by height in meters squared (kg/m²):

FFMI = Fat-Free Mass (kg)/(Height (m)) ².

FMI = Fat Mass (kg)/(Height (m)) ².

### Assessment of other variables

Baseline sociodemographic data and lifestyle factors were collected via standardized interviewer-administered questionnaires^7^. Age (years) and education (years) were recorded as continuous variables. Marital status was categorized as ‘Married’ versus ‘Not married’ (a group combining single, widowed, and divorced participants). Occupational status was dichotomized as ‘no’ (e.g., unemployed, homemaker, or not having a stable job) versus ‘yes’ (having a stable job).

Socioeconomic Status (SES) was determined using an asset-based index, a method commonly used in developing countries where income data may be unreliable^30^. The index was constructed based on ownership of household assets, including a refrigerator, freezer, washing machine, dishwasher, computer, mobile phone, car, and internet access, as well as home ownership and the number of rooms per person^30^. This composite index was treated as a continuous variable in all adjusted models.

Physical activity (Metabolic Equivalent of Task (MET)) levels were assessed using the validated International Physical Activity Questionnaire (IPAQ)^7^. Total physical activity was calculated and expressed as MET hours per week.

### Statistical analysis

Prior to statistical inference, comprehensive descriptive analyses were conducted to evaluate the distributions of the data. Histograms of all continuous variables were visually inspected to check for skewness and strong outliers. Variables exhibiting positive skewness and high outliers were addressed using logarithmic transformation to approximate normality. Additionally, frequency counts were generated for all categorical variables to identify and address any small categories. Descriptive characteristics of the final sample were then evaluated presented across gender-specific FFMI tertiles (T1–T3).

Continuous variables were analysed using independent-samples t-tests (for two-group comparisons) and one-way analysis of variance (ANOVA; for comparisons across tertiles), while categorical variables were assessed with chi-square tests. Age-adjusted comparisons were performed using analysis of covariance (ANCOVA).

Associations between dietary pattern scores, Protein Density, and FFMI were examined using multivariable linear regression models stratified by gender. Two distinct modelling strategies were employed to address different research objectives. Primary Models (A1–A3) estimated the total association between dietary predictors and FFMI without adjusting for fat mass. Model A1 (Crude Model) was unadjusted; Model A2 (Basic Adjusted Model) was adjusted for age, total energy intake (kcal/day), and physical activity (MET-hours/week); and Model A3 (Fully Adjusted Model) was adjusted for all variables in Model A2 plus education (years), marital status, employment status, and socioeconomic status. Partitioning Models (multivariable linear regression adjusted for FMI) (B1–B3) served as a secondary, mechanistic analysis by adjusting for FMI to isolate the association between dietary predictors and FFMI independent of adiposity, thereby reflecting potential effects on body composition partitioning. These models followed a similar structure: Model B1 (Basic Partitioning Model) was adjusted for age and FMI; Model B2 (Adjusted Partitioning Model) added total energy intake and physical activity to Model B1; and Model B3 (Fully Adjusted Partitioning Model) added the sociodemographic covariates to Model B2. In these analyses, FMI was entered explicitly as a continuous covariate (rather than using a residual-based approach) to statistically condition the outcome on adiposity, thereby isolating the variation in FFMI that is independent of FM.

Mediation analyses (Models A4 and B4) were conducted using the PROCESS macro in SPSS with robust variance using 5,000 bootstrap samples to assess physical activity (MET-hours/week) as a mediator in the relationships between dietary predictors (Healthy pattern, Unhealthy pattern, Protein Density) and FFMI. This analysis was conducted using two different modelling strategies, stratified by gender. First, primary mediation models (Models C1-C3) were run with adjustments corresponding to those in the primary regression models (A1-A3). Second, partitioning mediation models (Models D1-D3) served as a secondary, mechanistic analysis, using an adjustment structure identical to the partitioning regression models (B1-B3). This approach was used to isolate the mediated association between dietary predictors and FFMI independent of adiposity, thereby reflecting potential effects on body composition partitioning. For all models, the significance of indirect effects was determined by the 95% Bootstrap Confidence Interval (Boot CI); mediation was considered present if the interval did not include zero and p-value < 0.05 was considered statistically significant. All statistical procedures were performed using IBM SPSS Statistics, version 30.0.

## Electronic Supplementary Material

Below is the link to the electronic supplementary material.

Supplementary Material 1

## Data Availability

Data supporting the findings of this study are available from the corresponding author upon reasonable request.
