# Supplementary material for: Dietary patterns and protein density associations with fat-free mass in a cross-sectional analysis of rural Iranian adults with overweight and obesity
Source: Sci Rep. 2026 Apr 24;16:18977. doi: 10.1038/s41598-026-49776-6 (PMC13275905; doi:10.1038/s41598-026-49776-6)

Supplementary Table 1: Multivariable Linear Regression Analysis of the Association between Dietary Patterns, Protein Density, and Fat-Free Mass Index (FFMI): Partitioning Models Adjusted for Fat Mass Index (FMI)

|  | Fat-Free Mass Index (FFMI) | | | | | | | | | |
| --- | --- | --- | --- | --- | --- | --- | --- | --- | --- | --- |
|  | Model B1 (Basic Partitioning) | | | | | | Model B2 (Adjusted Partitioning) | | Model B3 (Fully Adjusted Partitioning) | |
|  | β (95% CI) | | | P-value | | | β (95% CI) | P-value | β (95% CI) | P-value |
| **Male** |  |  |  | |  | |  | |  | |
| Healthy Dietary Pattern | 0.08 (-0.01, 0.17) | | | 0.081 | | | 0.09 (-0.00, 0.18) | 0.054 | 0.09 (-0.01, 0.19) | 0.077 |
| Unhealthy Dietary Pattern | 0.10 (<0.01, 0.21) | | | 0.039 | | | 0.10 (0.00, 0.20) | 0.051 | 0.01 (<0.01, 0.02) | 0.050 |
| Protein Density | 0.01 (0.00, 0.02) | | | 0.055 | | | 0.01 (<0.01, 0.02) | 0.007 | 0.01 (<0.01, 0.02) | 0.008 |
|  |  |  |  | |  |  |  |  |  |  |
| **Female** |  |  |  | |  | |  | |  | |
| Healthy Dietary Pattern | -0.04 (-0.11, 0.02) | | | 0.212 | | | -0.03 (-0.10, 0.03) | 0.276 | -0.02 (-0.09, 0.05) | 0.533 |
| Unhealthy Dietary Pattern | 0.07 (0.01, 0.13) | | | 0.020 | | | 0.06 (-.001, 0.12) | 0.055 | 0.05 (-0.01, 0.12) | 0.109 |
| Protein Density | 0.006 (-0.00, 0.01) | | | 0.187 | | | 0.01 (<0.01, 0.02) | 0.033 | 0.01 (0.00, 0.02) | 0.047 |
|  |  |  |  | |  |  |  |  |  |  |

Model B1: Adjusted for fat mass index (FMI) and age; Model B2: Additionally adjusted for total energy intake (kcal/day) and physical activity (MET); Model B3: Additionally adjusted for education (years), marital status, occupational status, and socio-economic status.

Supplementary Table 2: Mediation Analysis of Physical Activity (MET) on the Association Between Dietary Patterns and Fat-Free Mass Index (FFMI): Partitioning Models Adjusted for Fat Mass Index (FMI)

|  | Fat-Free Mass Index (FFMI) | | | | | | | | |
| --- | --- | --- | --- | --- | --- | --- | --- | --- | --- |
|  | Direct Effect | | | | | Indirect Effect(s) | | | |
|  | β | | SE | 95% CI | P-value | β | Boot SE | 95% Boot CI | |
| **Male /** **Healthy Dietary Pattern** | |  |  |  | |  | |  | |
| Model D1 (Basic Partitioning) | 0.09 | | 0.04 | <0.01, 0.18 | 0.047 | -0.01 | 0.006 | -0.02-(-0.0007) | |
| Model D2 (Adjusted Partitioning) | 0.09 | | 0.04 | -0.00, 0.18 | 0.053 | -0.01 | 0.006 | -0.02-(-0.001) | |
| Model D3 (Fully Adjusted Partitioning) | 0.09 | | 0.05 | -0.00, 0.19 | 0.074 | -0.007 | 0.005 | -0.02-0.003 | |
|  |  |  |  |  |  |  |  |  |  |
| **Female / Healthy Dietary Pattern** | |  |  |  | |  | |  | |
| Model D1 (Basic Partitioning) | -0.03 | | 0.03 | -0.10, 0.03 | 0.297 | -0.007 | 0.003 | -0.01-(-0.001) | |
| Model D2 (Adjusted Partitioning) | -0.03 | | 0.03 | -0.10, 0.02 | 0.276 | -0.007 | 0.003 | -0.01-(-0.001) | |
| Model D3 (Fully Adjusted Partitioning) | -0.02 | | 0.03 | -0.09, 0.05 | 0.539 | -0.001 | 0.002 | -0.007-0.004 | |
|  |  |  |  |  |  |  |  |  |  |
| **Male / Unhealthy Dietary Pattern** | |  |  |  |  |  |  |  |  |
| Model D1 (Basic Partitioning) | 0.10 | | 0.05 | <0.01, 0.20 | 0.044 | 0.003 | 0.006 | -0.007-0.01 | |
| Model D2 (Adjusted Partitioning) | 0.10 | | 0.05 | -0.00, 0.20 | 0.0509 | 0.003 | 0.006 | -0.008-0.01 | |
| Model D3 (Fully Adjusted Partitioning) | 0.10 | | 0.05 | <0.01, 0.21 | 0.045 | -0.003 | 0.006 | -0.01-0.008 | |
|  |  |  |  |  |  |  |  |  |  |
| **Female / Unhealthy Dietary Pattern** | |  |  |  |  |  |  |  |  |
| Model D1 (Basic Partitioning) | 0.06 | | 0.03 | <0.01, 0.12 | 0.046 | 0.01 | 0.005 | 0.002-0.02 | |
| Model D2 (Adjusted Partitioning) | 0.06 | | 0.03 | -0.00, 0.12 | 0.055 | 0.009 | 0.004 | 0.001-0.02 | |
| Model D3 (Fully Adjusted Partitioning) | 0.05 | | 0.03 | -0.01, 0.12 | 0.101 | 0.001 | 0.002 | -0.002-0.008 | |
|  |  |  |  |  |  |  |  |  |  |
| **Male /** **Protein Density** | |  |  |  | |  | |  | |
| Model D1 (Basic Partitioning) | <0.01 | | <0.01 | -0.00, 0.01 | 0.087 | 0.001 | 0.0008 | -0.0001-0.002 | |
| Model D2 (Adjusted Partitioning) | 0.01 | | <0.01 | <0.01, 0.02 | 0.007 | 0.001 | 0.001 | 0.0004-0.004 | |
| Model D3 (Fully Adjusted Partitioning) | 0.01 | | <0.01 | <0.01, 0.02 | 0.008 | 0.001 | 0.0008 | 0.00-0.003 | |
|  |  |  |  |  |  |  |  |  |  |
| **Female / Protein Density** | |  |  |  | |  | |  | |
| Model D1 (Basic Partitioning) | <0.01 | | <0.01 | -0.00, 0.01 | 0.219 | 0.0005 | 0.0005 | -0.0004-0.001 | |
| Model D2 (Adjusted Partitioning) | 0.01 | | <0.01 | <0.01, 0.02 | 0.032 | 0.001 | 0.0009 | 0.0003-0.003 | |
| Model D3 (Fully Adjusted Partitioning) | 0.01 | | <0.01 | 0.00, 0.02 | 0.041 | 0.0006 | 0.0006 | -0.0003-0.002 | |
|  |  |  |  |  |  |  |  |  |  |

FFMI, fat-free mass index; MET, metabolic equivalent of task; SE, standard error; Boot CI, bootstrap confidence interval. Model D1: Adjusted for fat mass index (FMI) and age; Model D2: Additionally adjusted for total energy intake (kcal/day); Model D3: Additionally adjusted for education (years), marital status, occupational status, and socio-economic status.

Supplementary Figure 1. Distribution of the Healthy and Unhealthy Dietary Pattern Factor Scores (N = 2,299).


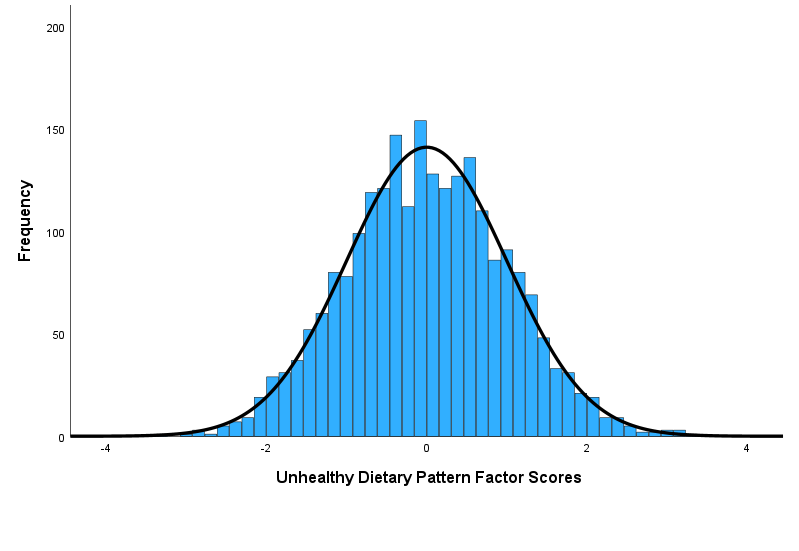

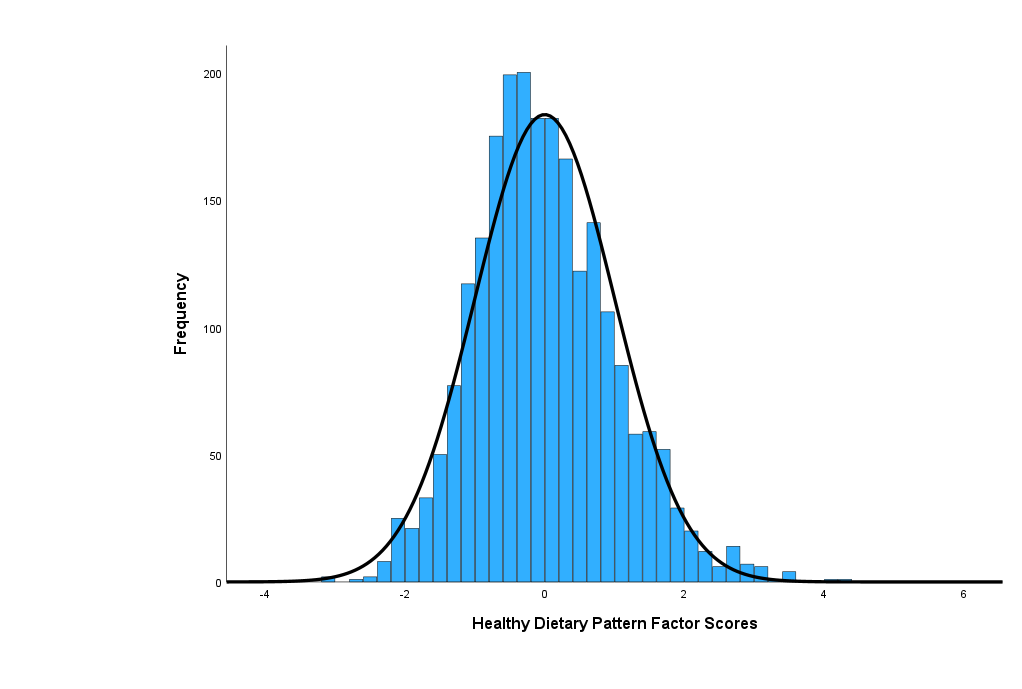

Supplement: Supplementary file 1 — Supplementary Material 1 [file 41598_2026_49776_MOESM1_ESM.docx]
